# Breast tissue, oral and urinary microbiomes in breast cancer

**DOI:** 10.18632/oncotarget.21490

**Published:** 2017-08-14

**Authors:** Hannah Wang, Jessica Altemus, Farshad Niazi, Holly Green, Benjamin C. Calhoun, Charles Sturgis, Stephen R. Grobmyer, Charis Eng

**Affiliations:** ^1^ Genomic Medicine Institute, Lerner Research Institute, Cleveland Clinic, Cleveland, OH, USA; ^2^ Cleveland Clinic Lerner College of Medicine, Cleveland Clinic, Cleveland, OH, USA; ^3^ Taussig Cancer Institute, Cleveland Clinic, Cleveland, OH, USA; ^4^ Surgical Oncology, Digestive Disease and Surgery Institute, Cleveland Clinic, Cleveland, OH, USA; ^5^ Department of Anatomic Pathology, Robert J. Tomsich Pathology and Laboratory Medicine Institute, Cleveland Clinic, Cleveland, OH, USA; ^6^ Comprehensive Breast Cancer Program, Cleveland Clinic, Cleveland, OH, USA; ^7^ Department of Genetics and Genome Sciences, Case Western Reserve University School of Medicine, Cleveland, OH, USA; ^8^ Germline High Risk Focus Group, CASE Comprehensive Cancer Center, Case Western Reserve University School of Medicine, Cleveland, OH, USA

**Keywords:** microbiome, metagenomics, breast cancer, oral, urine

## Abstract

It has long been proposed that the gut microbiome contributes to breast carcinogenesis by modifying systemic estrogen levels. This is often cited as a possible mechanism linking breast cancer and high-fat, low-fiber diets as well as antibiotic exposure, associations previously identified in population-based studies. More recently, a distinct microbiome has been identified within breast milk and tissue, but few studies have characterized differences in the breast tissue microbiota of patients with and without cancer, and none have investigated distant body-site microbiomes outside of the gut. We hypothesize that cancerous breast tissue is associated with a microbiomic profile distinct from that of benign breast tissue, and that microbiomes of more distant sites, the oral cavity and urinary tract, will reflect dysbiosis as well. Fifty-seven women with invasive breast cancer undergoing mastectomy and 21 healthy women undergoing cosmetic breast surgery were enrolled. The bacterial 16S rRNA gene was amplified from urine, oral rinse and surgically collected breast tissue, sequenced, and processed through a QIIME-based bioinformatics pipeline. Cancer patient breast tissue microbiomes clustered significantly differently from non-cancer patients (*p*=0.03), largely driven by decreased relative abundance of *Methylobacterium* in cancer patients (median 0.10 vs. 0.24, *p*=0.03). There were no significant differences in oral rinse samples. Differences in urinary microbiomes were largely explained by menopausal status, with peri/postmenopausal women showing decreased levels of *Lactobacillus*. Independent of menopausal status, however, cancer patients had increased levels of gram-positive organisms including *Corynebacterium* (*p*<0.01), *Staphylococcus* (*p*=0.02)*, Actinomyces* (*p*<0.01), and Propionibacteriaceae (*p*<0.01). Our observations suggest that the local breast microbiota differ in patients with and without breast cancer. Cancer patient urinary microbiomes were characterized by increased levels of gram-positive organisms in this study, but need to be further studied in larger cohorts.

## INTRODUCTION

Although breast cancer is the most common cancer in women worldwide [1], more than half of all women who develop the disease have no known risk factors [2, 3]. Moreover, only a fraction of those who harbor a genetic predisposition to breast cancer or who are exposed to known environmental risk factors go on to develop disease [3]. Clearly, additional contributing factors need to be identified. One such element that has garnered recent attention is the human microbiome, a dynamic community of bacteria, viruses, archaea, and eukaryotes that colonize human tissue [4]. The perturbation of these microbial communities, known as dysbiosis, has been linked not only to acute disease, but also to chronic diseases and malignancy [5–7]. Examples of this include the well-described role of *Helicobacter pylori* in gastric adenocarcinoma [8], and the emerging role of specific microbiomic profiles in colorectal cancer [9].

Microbiomic analysis of both breast milk and tissue shows that the human breast harbors unique and diverse microbiota [10, 11], one that is at least partially derived from translocation of gut microorganisms [12]. In addition to modulating the immune system, these gut microbes are known to play a vital role in estrogen metabolism; the perturbation of this “estrobolome” has been shown to influence systemic levels of estrogen and its metabolites [5, 7, 13, 14]. In fact, increased antibiotic use has been linked to increased risk of incident and fatal breast cancer in a case-controlled study of over 2,000 women [15]. Local estrogen levels, in addition to circulating ones, also play a role in breast carcinogenesis [16]. Malignant tissue in estrogen receptor (ER) positive breast cancer contains higher levels of estrogen metabolites as compared to normal breast tissue, attributed in part to altered intracrine signaling [17]. What remains unknown is the breast tissue microbiome’s role in local estrogen metabolism and breast carcinogenesis, and whether the microbiomes of any other body sites, outside of the gut, are affected by the systemic hormonal and immunologic disturbances associated with breast cancer.

Most prior work investigating the microbiome of breast tissue describes a community characterized by a predominance of the phyla Proteobacteria [10, 11] and Firmicutes [18], with one study finding a predominance of Bacteroidetes and very little Proteobacteria [19]. These investigations are still in their infancy, and the evidence also remains mixed on whether a difference exists between tumor and adjacent histologically-normal tissue from cancer patients. Additionally, these studies utilize a wide range of extraction methods, amplification primers, sequencers, bioinformatics pipelines, and patient populations. Additional studies are needed to elucidate which associations are strong enough to pursue functional studies. Furthermore, distant body-wide microbiomes, apart from the gut, have not yet been examined in breast cancer patients, and could represent a potentially non-invasive screening test.

In this study, we report on a prospectively accrued series of 57 patients with breast cancer and 21 healthy individuals undergoing breast surgery for cosmetic purposes. With these data, we correlate the microbiomic communities of breast tissue with breast cancer and its clinical-pathologic features in order to test the hypothesis that cancerous breast tissue has a distinct microbiome that is more pronounced in more aggressive disease, and that tumors of specific molecular subtypes may be associated with specific patterns as well. Furthermore, we compare, for the first time, the microbiomes of the oral cavity and urinary tracts in women with and without breast cancer.

## RESULTS

### Study population

Seventy-eight patients were enrolled in this study protocol. Among these, 57 underwent mastectomy for biopsy-proven invasive breast carcinoma. The other 21 patients were non-cancer controls who underwent cosmetic procedures including bilateral reduction mammoplasty or mastopexy. For 13 breast cancer patients and 1 non-cancer patient, study tissue could not be obtained at the time of surgery, thus only their urine and oral rinse samples were used. For 2 breast cancer patients, urine and oral rinse samples were unable to be obtained, thus only their breast tissue samples were analyzed. Cancer patients had a significantly higher mean age compared to non-cancer patients (55 vs. 43, *p = 0.002*) [Table 1]. Furthermore, mean BMI (27 vs. 35, *p < 0.001*), race, menopausal status, and time of last drink/meal differed significantly between groups. Clinical-pathologic characteristics of the breast cancer patients are listed in Supplementary Table 1.

**Table 1:** Demographics of study patients

Variable	Cancer (N=57)	Non-cancer (N=21)	P value
Age (years)	55 ± 14	43 ± 14	0.002
BMI	27 ± 6	35 ± 8	< 0.001
Race			0.013
White	47 (82)	11 (52)	
Black	9 (16)	9 (43)	
Other	1 (2)	1 (5)	
Premenopausal	18 (32)	14 (67)	0.009
Smoking History			0.441
Current	4 (7)	0 (0)	
Past	23 (40)	7 (33)	
Never	30 (53)	14 (67)	
Alcohol Use			0.388
Frequent	23 (40)	5 (24)	
Occasional	19 (33)	8 (38)	
None	15 (26)	8 (38)	
Last Antibiotic Use^a^			0.105
< 1 month ago	8 (15)	3 (16)	
1 – 6 months ago	8 (15)	7 (37)	
> 6 months ago	39 (71)	9 (47)	
Unknown	2	2	
			

Values are presented as means ± standard deviations or number (percent).

^a^ Data are missing for last antibiotic use for 4 patients. In these cases, percentages are calculated from denominator of samples with known data.

### Breast tissue microbiome

Depth of coverage was set to 60 sequences or higher based on leveling off of the Shannon diversity index at 60 reads. Due to this cutoff, a total of 39 cancer (17 tumor, 22 normal) and 24 non-cancer samples were included in the final analysis. Read counts were not significantly different in tumor (median 119), histologically normal (median 164), and non-cancer (median 379) samples (*p* = 0.12). Environmental controls, reagent, and no template controls clustered distinctly from patient samples by Principal Coordinates Analysis (PCoA) testing on unweighted UniFrac distances [Supplementary Figure 1].

**Figure 1:**
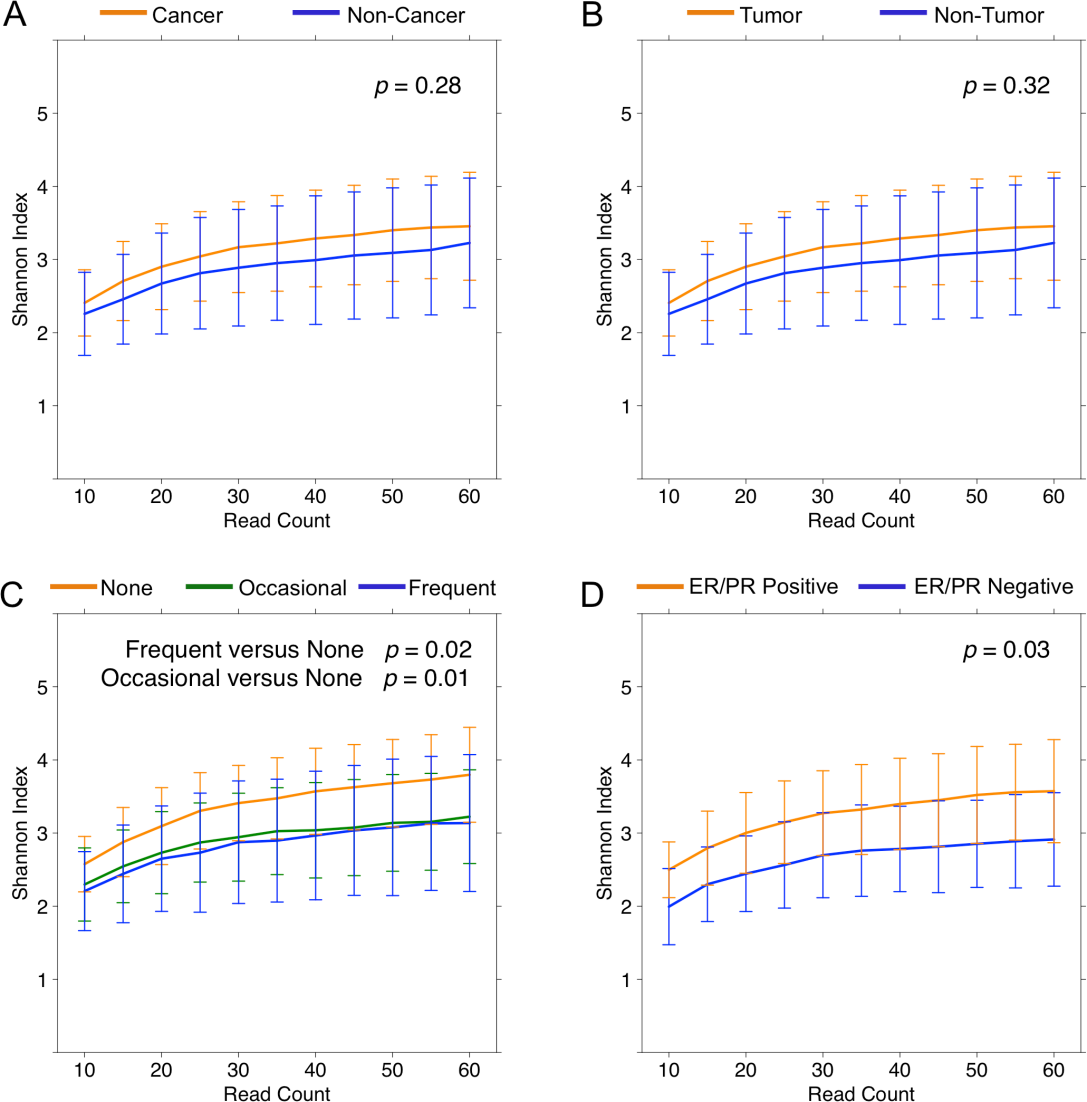
Alpha diversity rarefaction curves for breast tissue samples Rarefaction curves of Shannon diversity index up to 60 reads in **(A)** cancer (orange) and non-cancer (blue) samples, **(B)** tumor (orange) and non-tumor (blue) samples from cancer patients, **(C)** self-reported frequent (blue), occasional (green), and none (orange) alcohol users, and **(D)** hormone receptor positive (orange) and hormone receptor negative (blue) samples. Error bars represent standard deviation.

We found no significant differences in mean overall diversity as measured by Shannon diversity index (H), or species richness as measured by number of observed OTUs (N) between tissue from cancer (H = 3.5 ± 0.7, N = 20.3 ± 6.4) and non-cancer (H = 3.2 ± 0.9, N = 19.6 ± 7.0) patients (*p* = 0.28, 0.66) [Figure 1A]. We also found no significant difference between tumor (H = 3.6 ± 0.6, N = 20.3 ± 4.7) and histologically normal (H = 3.3 ± 0.8, N = 20.4 ± 7.7) samples from cancer patients (*p* = 0.32, 0.98) [Figure 1B]. However, self-reported non-alcohol consumers had significantly higher diversity: Frequent vs. None (H = 3.1 ± 0.9 vs. 3.8 ± 0.6, *p* = 0.02; N = 18.7 ± 8.0 vs. 23.9 ± 5.9, *p* = 0.03), as well as Occasional vs. None (H = 3.2 ± 0.6 vs. 3.8 ± 0.6, *p* = 0.01; N = 18.1 ± 4.1 vs. 23.9 ± 5.9, *p* < 0.01) [Figure 1C]. Additionally, among cancer patients, hormone receptor positive (H = 3.6 ± 0.8) samples had higher Shannon diversity relative to hormone receptor negative (H = 2.9 ± 0.6) samples (*p* = 0.03) [Figure 1D], but this only trended towards significance in the observed OTU count: hormone receptor positive vs. negative (N = 21.2 ± 6.4 vs. 16.5 ± 4.4, *p* = 0.08). In contrast, neither Shannon index nor observed OTU count significantly differed based on race, menopausal status, smoking history, last antibiotic use, last drink/meal, patient presentation, pathologic T-stage, focality, histologic subtype, histologic grade, *HER2* amplification status, lymphovascular invasion, or node positivity.

**Figure 2:**
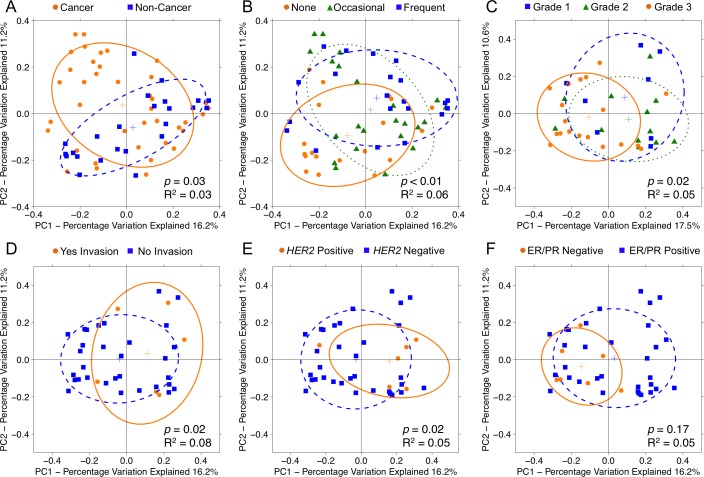
Principal coordinates analysis plots on unweighted UniFrac distances of breast tissue samples Overall oral microbiomic diversity of patient samples as represented by the first two principal coordinates on principal coordinates analysis of unweighted UniFrac distances. Each point represents a single sample, with plus sign and ellipses representing the fitted mean and 68% confidence interval of each group respectively. **(A)** Cancer samples (orange) clustered distinctly relative to non-cancer samples (blue), and **(B)** patient samples clustered by degree of alcohol use: none (orange), occasional (green), frequent (blue). Among cancer patients, samples clustered by **(C)** histologic grade: grade 1 (blue), grade 2 (green), grade 3 (orange), **(D)** presence of lymphovascular invasion: yes (orange), no (blue), **(E)** *HER2* amplification status: positive (orange), negative (blue), and **(F)** hormone receptor status: ER/PR negative (orange), ER/PR positive (blue). For hormone receptor status, only weighted UniFrac distance comparisons were significant (*p* = 0.05, R^2^ = 0.05), but the unweighted plot is presented here for consistency.

To test whether overall bacterial taxa composition was different between cancer and non-cancer tissue, we used principal coordinates analysis (PCoA) on unweighted UniFrac distances. We found that although the samples from cancer patients clustered significantly differently from those of non-cancer patients (*p* = 0.03, R^2^ = 0.03), the effect size was quite modest [Figure 2A]. Likewise, the distribution of unweighted UniFrac distances trended towards significance among patient samples from the same group vs. patient samples from different groups (*p* = 0.11) [Supplementary Figure 2]. Patient samples also clustered significantly by alcohol use (*p* < 0.01, R^2^ = 0.06), but not by any other demographic factors such as age, BMI, race, smoking history, menopausal status, or last antibiotic use [Figure 2B]. Among cancer patients, tumor and histologically normal samples did not cluster distinctly (*p* = 0.92). However, samples did cluster by histologic grade (*p* = 0.02, R^2^ = 0.05) [Figure 2C], presence of lymphovascular invasion (*p* = 0.02, R^2^ = 0.08) [Figure 2D], *HER2* amplification status (*p* = 0.02, R^2^ = 0.05) [Figure 2E]. In addition, samples clustered significantly by hormone receptor status on weighted (*p* = 0.05, R^2^ = 0.05), but not unweighted (*p* = 0.17), UniFrac distances [Figure 2F]. Samples did not cluster by patient presentation, pathologic T-stage, tumor size, focality, histologic subtype, or node positivity.

**Figure 3:**
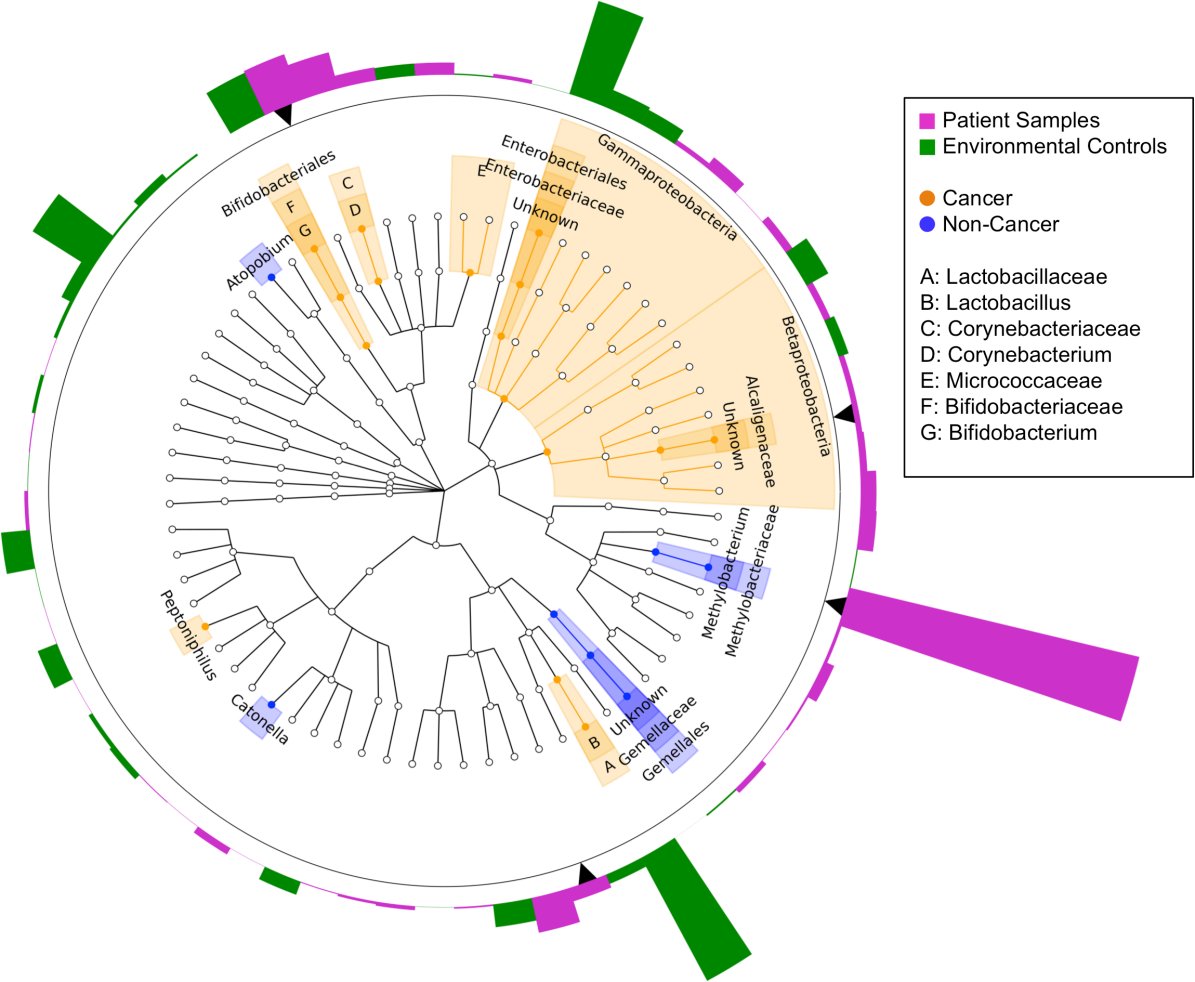
Cladogram of differentially abundant taxa in cancer and non-cancer patient breast tissue Cladogram depicting phylogenetic relationship of taxa identified as significantly different (*p* < 0.05) by Wilcoxon rank-sum testing in cancer as compared to non-cancer patient samples. Each concentric ring of nodes represents a taxonomic rank, starting with phylum and ending with genus. Nodes highlighted in orange are increased in cancer relative to non-cancer samples, and nodes highlighted in blue are increased in non-cancer relative to cancer samples. Each bar in the circular bar plot surrounding the cladogram represents the difference in mean relative abundance of each genus in patient samples as compared to environmental controls, with a greater height indicating a larger difference. The color of the bars indicates the direction of the difference: higher in environmental samples (green), higher in patient samples (magenta). Black arrows indicate genera identified as significantly increased in patient samples as compared to environmental controls. Only *Methylobacterium* and an unknown genus of the family Alcaligenaceae were significantly differentially abundant in cancer and non-cancer samples, in addition to being significantly increased in patient samples above environmental controls.

**Figure 4:**
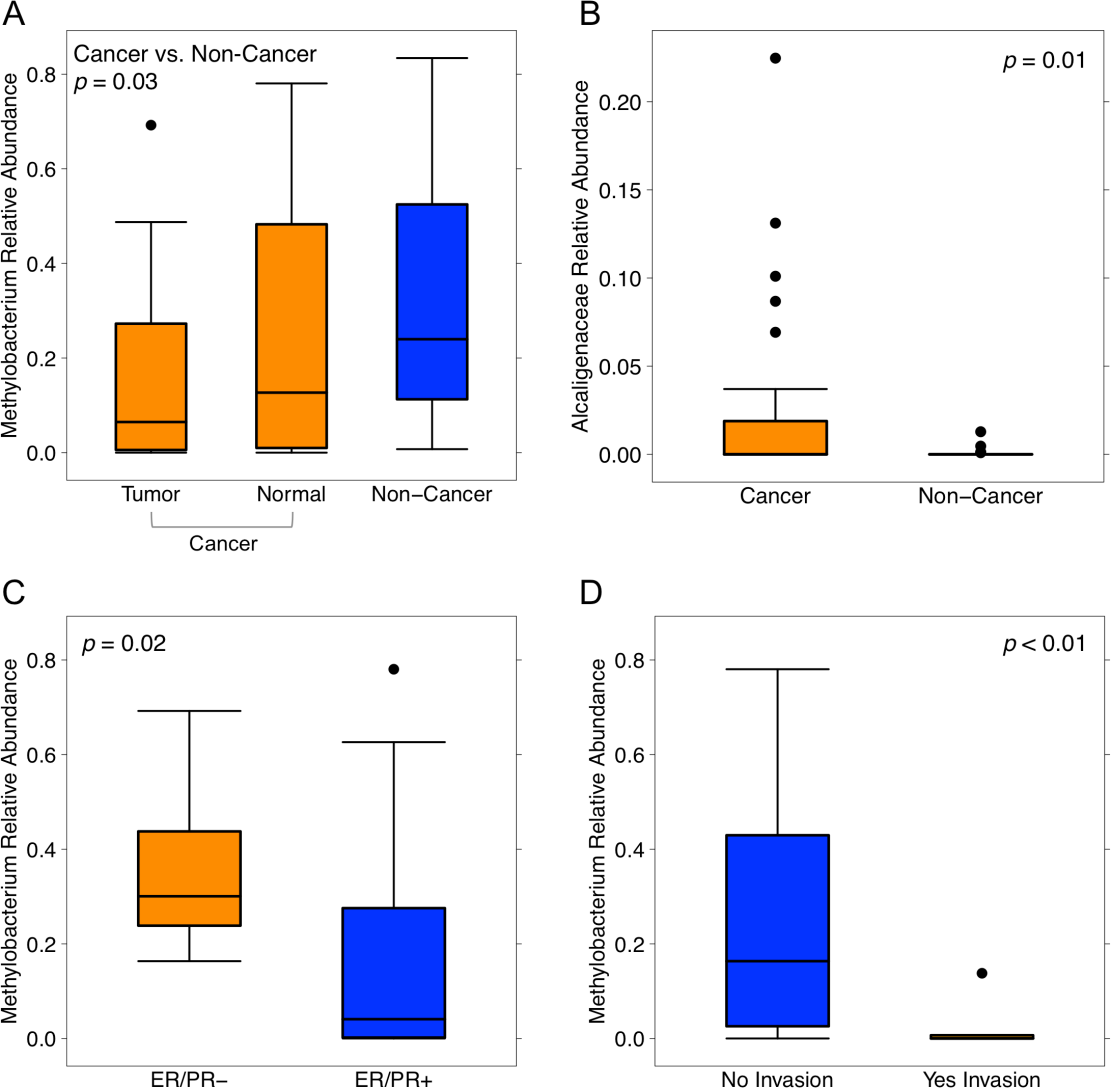
Relative abundances of Methylobacterium and Alcaligenaceae by sample type and clinical-pathologic features Box plots representing **(A)** relative abundances of genus *Methylobacterium* by sample type: cancer (orange) and non-cancer (blue), **(B)** relative abundances of unknown genus of family Alcaligenaceae by sample type: cancer (orange), and non-cancer (blue), relative abundances of genus *Methylobacterium* by **(C)** hormone receptor status: ER/PR negative (orange), ER/PR positive (blue), and **(D)** lymphovascular invasion: no (blue), yes (orange). Dark horizontal lines represent the median, with the box representing the first (Q1) and third (Q3) quartiles, the outer fences representing 1.5 x interquartile range, and the black circles representing outliers.

Next, we compared the relative abundances of individual taxa between cancer and non-cancer patient samples, finding increased relative abundance of 6 genera and 2 classes, and decreased relative abundance of 5 genera in cancer relative to non-cancer patients, out of a total of 165 taxa [Figure 3]. Of these differentially abundant taxa, only 2 genera were found to be significantly increased in patient relative to environmental control samples: genus *Methylobacterium* and an unknown genus of family Alcaligenaceae. *Methylobacterium* was decreased in cancer relative to non-cancer patient samples (median 0.10 vs. 0.24, *p* = 0.03). Additionally, although not statistically significant, histologically normal tissue from cancer patients had higher relative abundance of *Methylobacterium* compared to tumor tissue, but lower abundance compared to non-cancer patient tissue [Figure 4A]. Alcaligenaceae was increased in cancer relative to non-cancer patient samples (median 0.00 vs. 0.00, *p* = 0.01), predominantly driven by high relative abundances in the top quartile of cancer patients (Q3-Q4 0.02-0.22 vs. 0.00-0.01) [Figure 4B].

Lastly, relative abundances of *Methylobacterium* and Alcaligenaceae were not associated with any demographic features including age, BMI, race, smoking history, alcohol use, menopausal status, or last antibiotic use. While Alcaligenaceae was not associated with any clinical-pathologic features listed in Table 1, *Methylobacterium* was significantly decreased in hormone receptor-positive samples compared to hormone receptor-negative samples (median 0.04 vs. 0.30, *p* = 0.02), and in samples with histo-pathologic evidence of lymphovascular invasion versus those without (median 0.00 vs. 0.16, *p* < 0.01) [Figure 4C–4D].

### Oral rinse microbiome

Depth of coverage for oral rinse samples was set to 20,000 sequences or higher based on this being the cutoff between environmental control samples and patient samples. All patient samples (55 cancer, 21 non-cancer) were included in the analysis. Read counts were not significantly different in cancer (median 156,834) and non-cancer (median 152,922) patients (*p* = 0.52). We found no significant differences in mean measures of alpha diversity, overall community structure by PCoA, or relative abundances of individual taxa in oral rinses of cancer and non-cancer patients [Supplementary Figure 3].

### Urine microbiome

Depth of coverage for urine samples was set to 10,000 sequences or higher based on this being the cutoff between environmental control samples and patient samples. Due to this cutoff, a total of 46 cancer patient samples and 19 non-cancer samples were included in the analysis. Read counts were not significantly different in cancer (median 125,037) and non-cancer (median 203,842) patients (*p* = 0.15).

**Figure 5:**
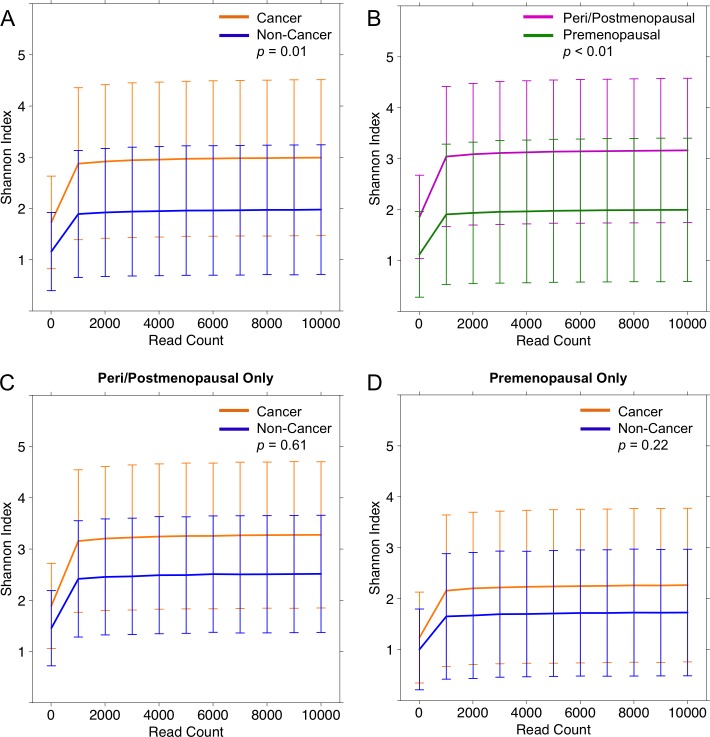
Alpha diversity rarefaction curves for urine samples Rarefaction curves of Shannon diversity index up to 1000 reads in **(A)** cancer (orange) and non-cancer (blue) samples and **(B)** peri/postmenopausal (magenta) and premenopausal (green) samples from cancer patients. When stratified by menopausal status, Shannon index was no longer significantly different in cancer and non-cancer samples, in either **(C)** peri/postmenopausal patient samples: cancer (orange), non-cancer (blue), or **(D)** premenopausal patient samples: cancer (orange), non-cancer (blue). Error bars represent standard deviation.

We observed that cancer patients (H = 3.0 ± 1.5) had significantly higher Shannon diversity relative to non-cancer patient (H = 2.0 ± 1.3) urine samples (*p* = 0.01) [Figure 5A], but this only trended towards significance in the observed OTU count: cancer vs. non-cancer (N = 268.7 ± 126.2 vs. 211.4 ± 71.8, *p* = 0.07). However, menopausal status was also an important predictor of urine microbial diversity, with peri/postmenopausal samples (H = 3.2 ± 1.4) being more diverse than premenopausal samples (H = 2.0 ± 1.4) (*p* < 0.01) [Figure 5B]. Again this trended towards significance in the observed OTU count: peri/postmenopausal vs. premenopausal (N = 273.0 ± 131.3, N = 220.2 ± 78.0, *p* = 0.07). When subset by menopausal status, cancer samples continued to have higher diversity relative to non-cancer patients, in both peri/postmenopausal and premenopausal subgroups, although this difference was no longer statistically significant [Figure 5C–5D]. No other demographic or clinical-pathologic features were associated with differences in alpha diversity.

**Figure 6:**
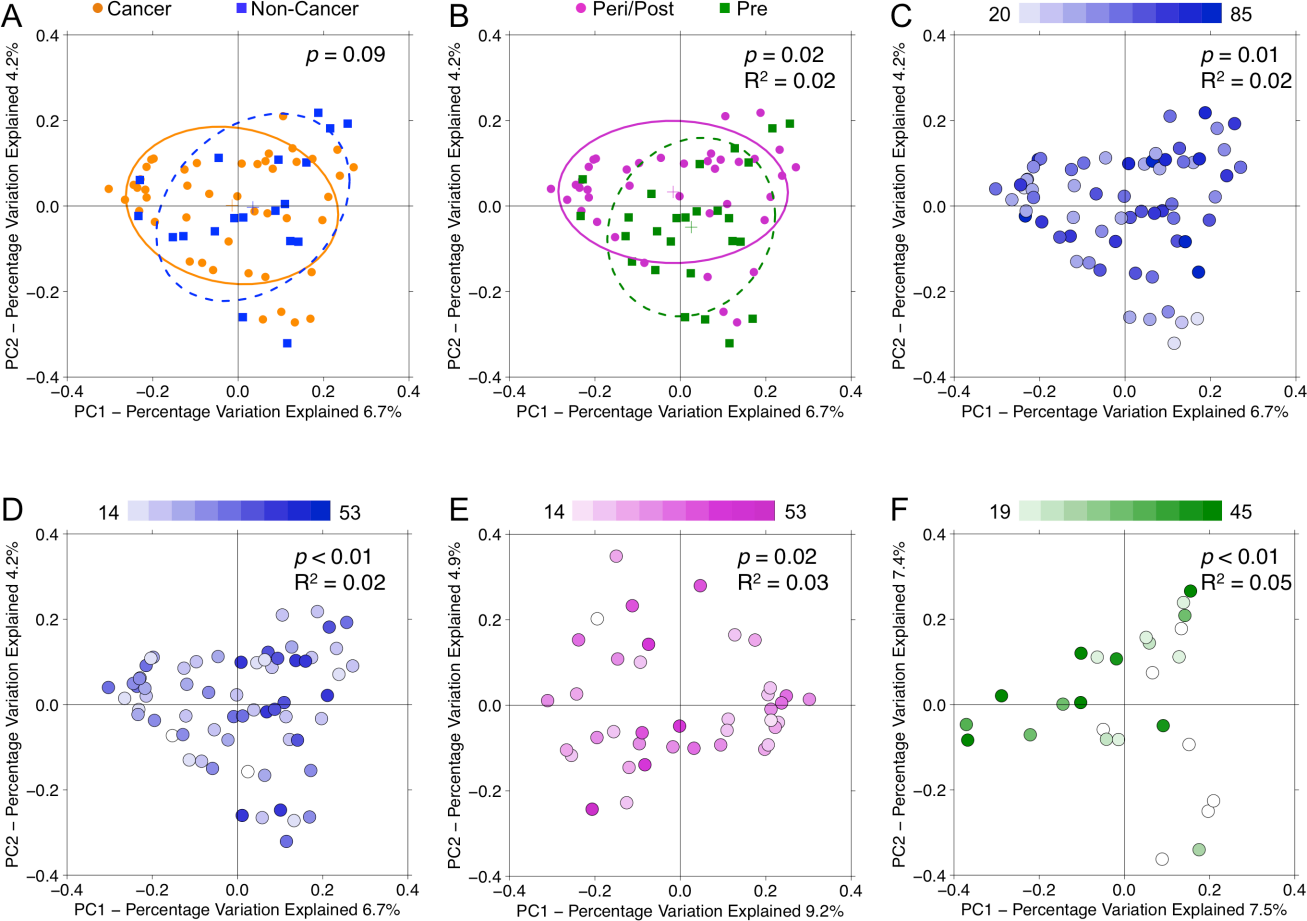
Principal coordinates analysis plots on unweighted UniFrac distances of urine samples Overall oral microbiomic diversity of patient samples as represented by the first two principal coordinates on principal coordinates analysis of unweighted UniFrac distances. Each point represents a single sample, with plus sign and ellipses representing the fitted mean and 68% confidence interval of each group respectively. While **(A)** cancer (orange) and non-cancer (blue) samples did not cluster distinctly, **(B)** peri/postmenopausal (magenta) and premenopausal (green) samples did cluster significantly differently. Additionally, urine samples separated by **(C)** age (younger to older: white to blue) and **(D)** BMI (younger to older: white to blue). Even when stratifying by menopausal status, urine samples continued to separate significantly by BMI in both **(E)** peri/postmenopausal and **(F)** premenopausal patients.

Analysis of beta diversity by PCoA demonstrated that samples from cancer and non-cancer patients were not significantly different from one another (*p* = 0.09) [Figure 6A]. Urine samples clustered significantly by menopausal status (*p* = 0.02, R^2^ = 0.02), age (*p* = 0.01, R^2^ = 0.02), and BMI (*p* < 0.01, R^2^ = 0.02), but not by other demographic factors [Figure 6B–6D]. When subset by menopausal status, BMI continued to account for significant clustering in both peri/postmenopausal (*p* = 0.02, R^2^ = 0.03) and premenopausal (*p* < 0.01, R^2^ = 0.05) samples [Figure 6E–6F], but age did not. Among cancer samples, we observed no significant clustering by any clinical-demographic features.

**Figure 7:**
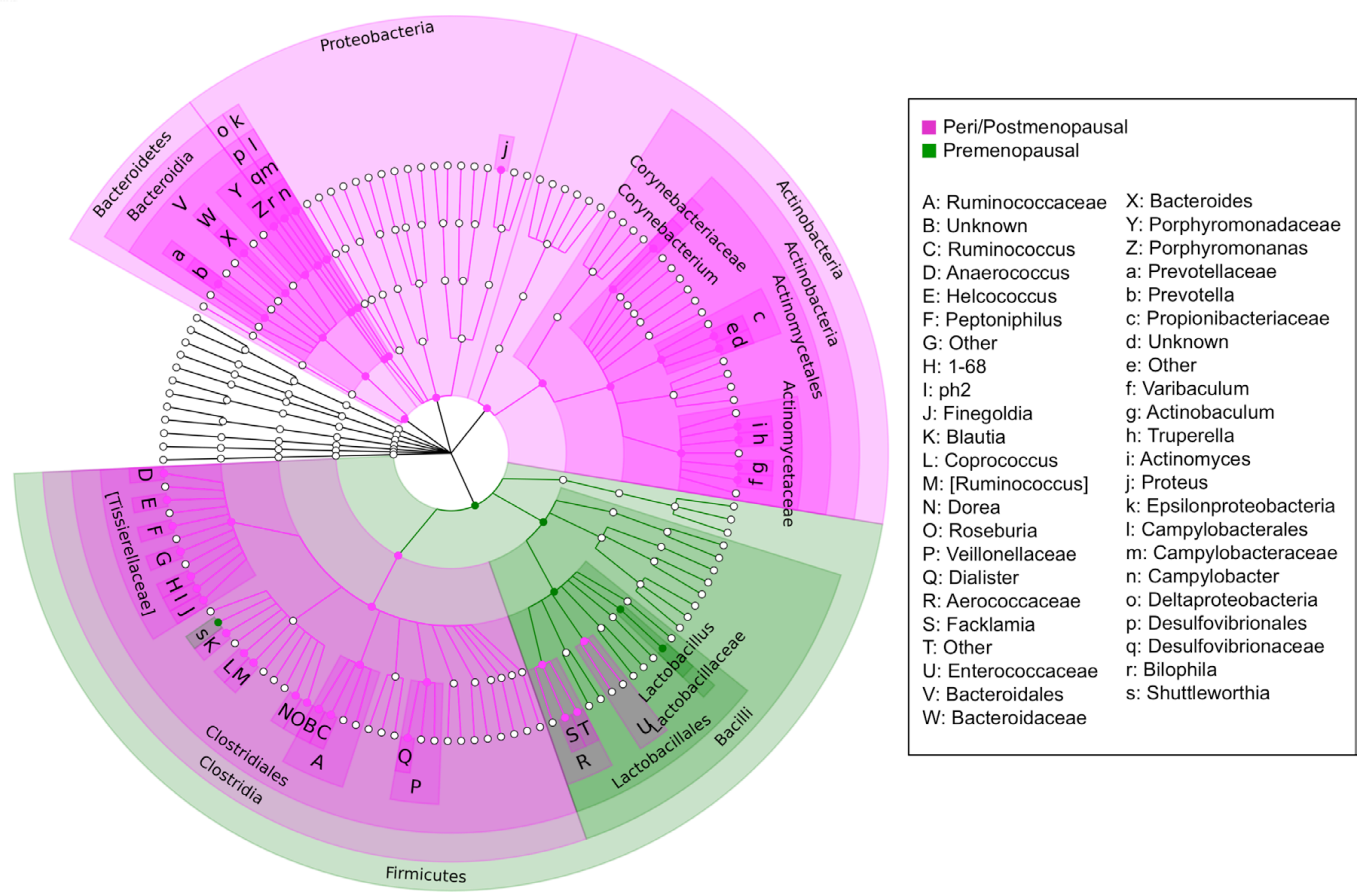
Cladogram of differentially abundant taxa in peri/postmenopausal and premenopausal patient urine Cladogram depicting phylogenetic relationship of taxa identified as significantly different (*p* < 0.05) by Wilcoxon rank-sum testing in peri/postmenopausal and premenopausal patient urine samples. Each concentric ring of nodes represents a taxonomic rank, starting with phylum and ending with genus. Nodes highlighted in magenta are increased in peri/postmenopausal relative to premenopausal samples, and nodes highlighted in green are increased in premenopausal relative to peri/postmenopausal samples. The urine samples of peri/postmenopausal women is characterized by a loss of *Lactobacillus*, and a concomitant increase in taxa from most other phyla, particularly anaerobes.

**Figure 8:**
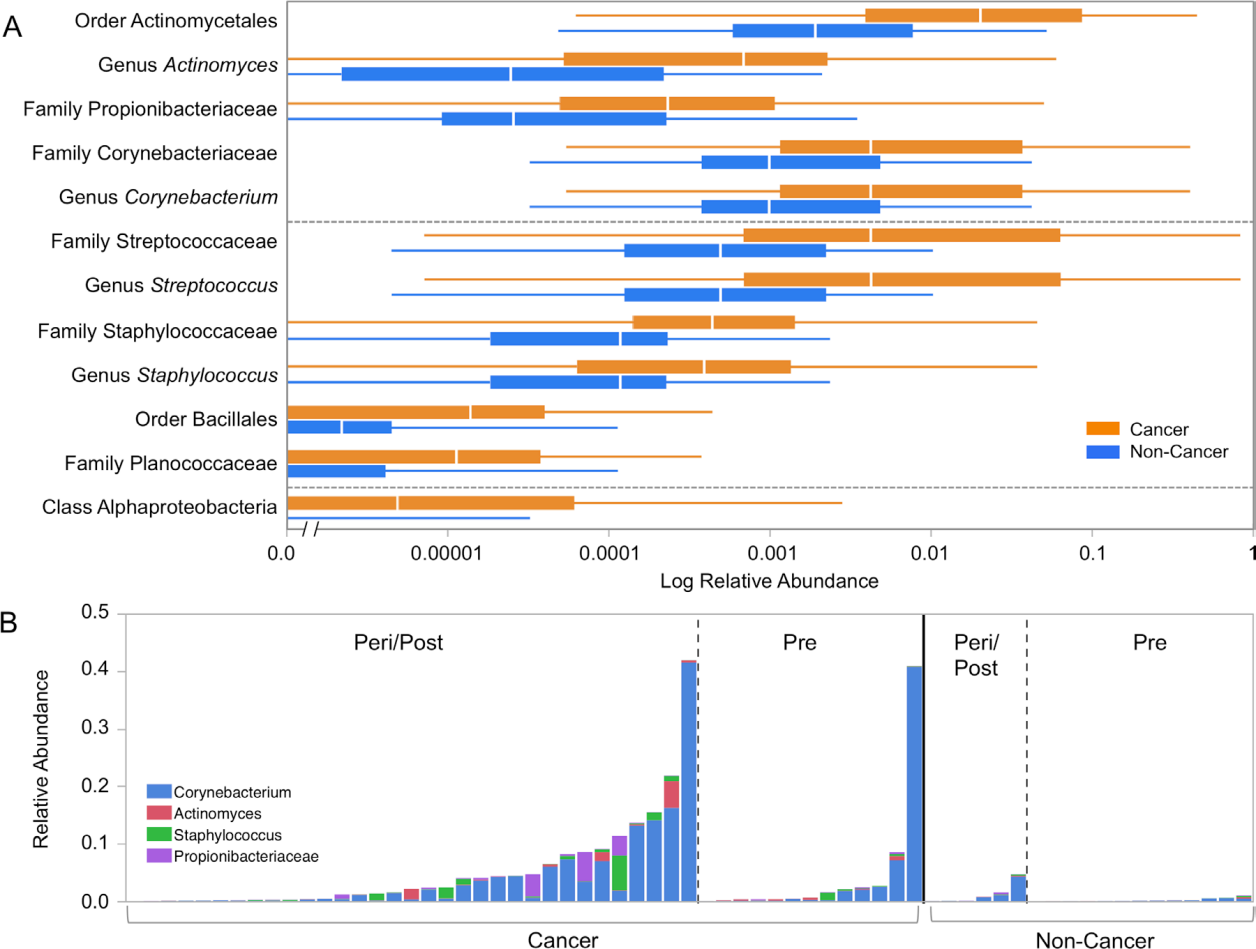
Relative abundances of differentially abundant taxa in cancer and non-cancer patient urine **(A)** Box plots representing log relative abundances of taxa identified as significantly different (*p* < 0.05) by Wilcoxon rank-sum testing in cancer (orange) and non-cancer (blue) patient urine samples. Note that the x-axis is plotted on a logarithmic scale, with an axis break to allow for plotting of zero-values. **(B)** Stacked bar graph representing relative abundances of *Corynebacterium* (blue), *Actinomyces* (red), *Staphylococcus* (green), and Propionibacteriaceae (purple) in individual samples grouped by menopausal and cancer status. *Streptococcus* and Planococcaceae were not represented due to being significantly correlated with BMI [Supplementary Figure 3].

Menopausal status was the largest driver of differences in relative abundances of individual taxa in our cohort. The microbiome of urine from peri/postmenopausal women was characterized by decreased abundance of genus *Lactobacillus*, and increased abundances of numerous other genera, including but not limited to anaerobes such as *Varibaculum*, *Porphyromonas*, *Prevotella*, *Bacteroides*, and members of the class Clostridia [Figure 7]. Using LEfSe, we identified 4 genera, 5 families, 2 orders, and 1 class (out of 258 total taxa compared) that were significantly increased in cancer relative to non-cancer patient urine samples after controlling for menopausal status by subsetting [Figure 8A–8B]. These differences were further confounded by the significant influence of BMI on taxa relative abundances as demonstrated by PCoA; higher BMI was associated with decreased genus *Streptococcus* (*p* = 0.05, R^2^ = 0.06) and family Planococcaceae (*p* < 0.01, R^2^ = 0.31) [Supplementary Figure 4]. Overall, we observed increased levels of *Corynebacterium*, *Staphylococcus*, *Actinomyces*, and Propionibacteriaceae in cancer relative to non-cancer samples, independent of both menopausal status and BMI.

## DISCUSSION

In this study, we sought to describe the microbiome of individuals with breast cancer, and to compare their microbiomes at different body sites with healthy controls. We hypothesized that breast carcinoma tissue would have a microbiome unique from that of surrounding normal parenchyma, that hormone receptor status of the carcinoma would be associated with microbes able to metabolize estradiol/progesterone, and that breast cancer patients would have altered microbiomes relative to healthy controls, both at the local tissue level and at more distant sites. The simple comparison of invasive carcinoma and paired normal tissue revealed no major shifts in overall diversity or microbiomic content. Similarly, oral rinse samples in cancer and non-cancer patients showed no large-scale differences. Notably, however, analysis of breast tissue revealed that cancer and non-cancer patients had significantly different microbiomes, a difference characterized largely by the decreased abundance of *Methylobacterium* in cancer patients. Urine sample analysis showed that while the biggest microbiomic differences were due to menopausal status, cancer patients harbor urinary microbiomes with increased abundance of gram-positive bacteria associated with skin flora.

The observation that the overall diversity of tumor and paired normal tissue samples from breast cancer patients are largely similar imply that more similarities than differences exist between the overall breast tissue microbiomes of tumor and adjacent normal tissues from the same patient, consistent with Urbaniak et al.’s findings [18]. In contrast, another previous, smaller, study has suggested conflicting results when comparing tumor tissue to paired normal tissue in breast cancer patients [11]. Key differences may be that our tumor and “normal” tissue was histologically-verified at the time of extraction and reclassified, if warranted, and that we used flash-frozen aseptically collected tissue, as did Urbaniak et al. rather than formalin-fixed paraffin-embedded tissues. Still, our analyses may have been limited by being underpowered by both sample size and low read count, despite being larger than the previous. Due to risk of a false negative finding, larger studies will be needed in the future to confirm these findings. At least one other recent study, using fresh intraoperatively-collected tissue, has found tumor and adjacent benign breast tissue to be largely similar as well [18]. If adjacent benign breast tissue is truly more similar to that of carcinoma tissue than to breast tissue from women without cancer, it may imply a breast-wide predisposition to carcinogenesis. Alternatively, these similarities may suggest that the microbial differences observed are secondary to the effect of the tumor rather than a causative agent.

Breast tissue is an inherently “low-biomass” body site, and the read counts obtained in this study are low compared to prior studies [10, 11]. This may be due to the fact that we used MO BIO extraction kits, and took precautions to keep samples in aseptic conditions until sequencing. MO BIO extraction kits have been shown to have the least contamination compared to other commercially available microbiome-focused DNA extraction kits [20]. Additionally, in an effort to minimize non-specific amplification, we size-selected the amplicon using gel-purification and limited the number of amplification cycles to 25. We acknowledge that the power to detect statistically significant differences in relative abundances is limited by the low read counts in this study. As such, the likelihood of false negatives in this study is quite high. However, there is evidence to suggest that useful comparisons can be made at this sequencing depth [21]. Indeed, prior studies have demonstrated that low numbers of reads can accurately characterize communities at the phylum-level, and be used to uncover large-scale differences between communities through analysis of beta-diversity metrics [22–24].

Despite sample size and read count limitations, we found that the breast tissue microbiomes of cancer and non-cancer patients were significantly different from each other by the measure of UniFrac distances, albeit with modest effect size. While age, BMI, race, and menopausal status were significantly different in the cancer and non-cancer groups, they did not significantly contribute to variation in the PCoA plots. This suggests that the differences seen on beta diversity analysis are due to the cancer versus non-cancer groupings, not to demographic differences. Additionally, while PCoA identified differences based on hormone receptor and *HER2* status in cancer samples, we were unable to resolve individual taxa that explained these differences due to small sample size and high background noise.

The primary bacterial genus driving the cancer-versus-non-cancer breast tissue microbiomic separation is the genus *Methylobacterium*. *Methylobacterium* belongs to the phylum Proteobacteria, and while its main habitat is soil and water, certain species have been identified in the human oropharynx and foot [25, 26]. Indeed, Methylobacterium has been identified as a known environmental contaminant, especially in low-biomass samples [20]. However, we found that the levels of Methylobacterium detected in patient samples were far greater than levels detected in the environmental controls, making this finding unlikely to solely be due to environmental contamination. They are facultative methylotrophs, meaning that they are able to use methanol and methylamine as fuel, and have been shown to produce phytohormones like cytokinin and auxin [27]. In rare cases, they have been identified as human pathogens [28]. Some of these phytohormones have been described to exert an anti-cancer effect [29, 30]. While this is certainly in line with our data that *Methylobacterium* is highest in non-cancer patients and lowest at the site of the tumor, and that it is associated with tumors with greater invasive potential, it is premature to suggest that local depletion of *Methylobacterium* increases malignant potential. Indeed, a prior study suggests that *Methylobacterium radiotolerans* is increased at the site of tumor [11]. However, this may be due to a myriad of differences between our studies (use of flash-frozen vs. formalin-fixed paraffin embedded tissue, type of extraction kit, 16S rRNA primers), or a matter of species specificity. In contrast, the family Alcaligenaceae was increased in cancer relative to non-cancer samples. Several members of this family are known human pathogens, for example, various species of *Achromobacter* and *Bordetella*, especially in immunocompromised hosts [31].

The secondary aim of our study was to identify possible sites of dysbiosis distant from the breast tissue that might serve as a non-invasive biomarker of breast carcinoma. Previous work in this area has concentrated on the gut microbiome. In 2011, Plottel et al. [7] extensively discussed the estrobolome, consistent of the enteric bacterial genes whose products metabolize estrogen and its metabolites. Perturbations in this collection of microbiota (e.g., via antibiotics) can lead to elevated levels of circulating estrogens, thereby increasing the risk of breast cancer. Indeed, clinical studies have identified associations between the gut microbiome and urinary estrogens and estrogen metabolites [13, 14]. Recently, a case-control study of 48 post-menopausal breast cancer patients and 48 controls found the gut microbiome to be less diverse and compositionally distinct in the women with breast cancer [32]. Supporting this hypothesis, antibiotic use has been found to be associated with the risk of incident and fatal breast cancer in a dose-dependent matter [15].

While we did not investigate the gut microbiome in this study, as stool samples would have required an additional visit and inconvenience to our patients, we did examine more easily accessible sites such as the oral rinse and urinary microbiomes. In the oral rinse microbiome, we found no differences between cancer and non-cancer patients despite epidemiologic data suggesting that periodontal disease was associated with increased breast cancer risk [33]. This is most likely due to the fact that the oral cavity is exposed to the environment through vocalization, air-exchange, and food and fluid ingestion on a constant basis. In fact, our data showed that relative abundances of Streptophyta and *Streptococcus* were significantly increased in patients who reported oral intake in the hour prior to their oral rinse. The levels of Streptophyta likely reflect chloroplasts from ingested plant material [34]. The relative increase in *Streptococcus* may be due to the ingestion of carbohydrates [35]. Thus, the microbes at this site are likely less permanent and more heavily influenced by the environment relative to internal factors than at sites like in the tissue and urinary tract.

Additionally, it is unclear whether oral rinse is the best method by which to capture the microbiota of the oral cavity; other studies have used swabs or saliva. Although there are likely no large differences in oral microbiome composition of breast cancer and non-cancer patients based on the negative findings in our study, we cannot exclude the possibility of small to moderate difference in microbial communities that would be detected in a larger cohort. While the way in which we collected the oral rinse samples most closely approximates what would be feasible for a non-invasive screening test in a clinical setting, our study is limited by the lack of control for diet and temporal variation. As such, there exists the risk of false negative findings in this study. Future studies on the oral microbiome in breast cancer should ideally control for oral intake and evaluate multiple samples from the same patient over a 24 hour period.

In the urine, we found microbiomic differences driven not only by the cancer versus non-cancer designation, but also by menopausal status and BMI. While increased overall diversity is associated with health and decreased diversity with colorectal cancer in the context of the gut microbiome [36], the opposite is true of the urogenital tract. For example, the vaginal tract microbiome in premenopausal women has been described to be less diverse, characterized by a relative overabundance of *Lactobacillus* relative to postmenopausal women [37], and can be at least partially restored in patients taking exogenous estrogens [38, 39]. It is no surprise, then, that our urine results reflect this difference, although this is, to the authors’ knowledge, the first time it has been reported in the urine. These findings could be due to contamination with vaginal or skin flora at time of collection (despite mid-stream clean catch protocol), or to colonization of the urethra and bladder by vaginal or skin flora [40]. This caveat may be less plausible because we cannot find a systematic reason why vaginal or skin flora contamination might affect only one group of patients.

Additionally, although overall diversity and community structure were not significantly different in cancer and non-cancer patients after sensitivity analysis, we observed changes in relative abundance of several taxa independent of menopausal status and BMI. Specifically, *Corynebacterium*, *Staphylococcus*, *Actinomyces*, and Propionibacteriaceae are all gram-positive microbes that are generally associated with skin flora (with the exception of *Actinomyces*). Gram-positive bacteria are known to be strong inducers of interleukin-12 (IL-12), as opposed to gram-negative bacteria, which preferentially stimulate IL-10. IL-12 goes on to promote interferon gamma (IFN-γ) secretion from T and NK cells, ultimately activating cytotoxic effects [41]. While IFN-γ is classically associated with anti-tumor effects, recent literature has shown that in the right context, IFN-γ can induce the opposite effect by upregulating proliferative signals and allowing tumor cells to escape recognition by cytotoxic T cells and NK cells [42, 43]. Colonization of the urogenital tract by gram-positive bacteria, perhaps opportunistically during menopause when levels of protective *Lactobacillus* decline, could potentially lead to chronic low-level IFN-γ activity and escape of breast cancer cells from interferon regulation and tumor progression [44]. Moving forward, it will be necessary to establish a timeline of urinary dysbiosis relative to cancer diagnosis/progression; if this pattern persists in patients over time prior to cancer diagnosis and/or progression, urine screening may represent a non-invasive way to identify high-risk patients in the future.

While this is one of the largest studies to examine the microbiome in human breast cancer patients, this study is still limited by sample size, and is underpowered to detect taxa-level differences in relative abundance after multiple testing correction. As such, we did not implement any multiple testing corrections, increasing our risk for detection of false positive results in an effort to minimize false negative results. Additionally, our analyses comparing cancer and non-cancer patient breast tissue, oral rinse, and urine microbiomes are potentially confounded by the effects of age, BMI, race, and menopausal status. We elected not to conduct an adjusted analysis as we would have been severely underpowered to do so based on sample size. However, in breast tissue, comparisons on alpha and beta diversity metrics, as well as relative abundances of *Methylobacterium* and *Alcaligenaceae* were not significantly different when analyzed by age, BMI, race, and menopausal status. This suggests that the differences observed in breast tissue are not due to these potential confounders. In urine, our finding of no significant differences between cancer and non-cancer patients could have been due to confounding from significant differences by menopausal status and BMI. In the taxa-level analysis in urine, we adjusted for menopausal status by using LEfSe’s subclass function to essentially conduct sensitivity analysis, only identifying differentially abundant taxa in cancer and non-cancer groups within each menopausal class. While logistically challenging, future studies in this area should aim to match their cohorts on the basis of age, race, BMI, and menopausal status if possible.

Overall, our observations suggest that the microbiomes of tumor and paired benign tissue from breast cancer patients are largely similar in terms of overall diversity and microbiomic content. Notably, however, cancer patients had significantly different microbiomes compared to non-cancer patients in both the local breast tissue and in the urinary tract. The tissue microbiome of cancer patients is driven by a relative decreased abundance in the genus *Methylobacterium*, and the urinary microbiome of cancer patients is characterized by relatively increased levels of gram-positive bacteria independent of demographic factors such as menopausal status and BMI. The investigation of the microbiome in breast cancer patients is still in its infancy. Further validation with a larger cohort of clinically matched patients will be needed to determine the significance and biological relevance of these findings.

## MATERIALS AND METHODS

### Patient enrollment and sample collection

With approval from our Institutional Review Board for human subjects protection and after written informed consent, we planned to enroll 50 breast cancer patients and 20 healthy controls for this study from a multidisciplinary breast cancer center at a large academic hospital from 2014-2016. Breast cancer patients eligible for inclusion for this study were over 18 years of age, female, had tumors greater than or equal to 2 cm in size, and were undergoing mastectomy. The second 2 criteria were to allow for sufficient quantity of tissue to be obtained for this study. Patients receiving neo-adjuvant therapy prior to surgery or with active clinical breast infection were excluded from the study. Control patients eligible for inclusion were older than 18 years of age, female, and undergoing breast surgery for cosmetic reasons (reduction mammoplasty or mastopexy). Control patients with a past history of cancer were excluded from the study. Demographics, clinical history, and risk factors for these patients were collected prospectively at the time of enrollment through a combination of patient interview and medical chart review [Supplementary Table 2]. Missing data was filled in via retrospective chart review; individuals without available data were noted as such in Table 1.

From each breast cancer and each control patient, we obtained a midstream clean-catch urine sample and a saline oral rinse sample at the time of written consent and centrifuged at 600 x g for 10 minutes. After decanting the supernatant, the pellet was frozen and stored at −80°C until nucleic acid extraction. At the time of surgery, we intraoperatively and aseptically collected two tissue samples from the patient: right and left breast from each control patient, and tumor and ipsilateral adjacent normal breast tissue for each cancer patient. These samples were transported in sterile containers to the pathology lab, where they were dissected with sterile forceps and scalpels in a sterile hood to prevent contamination. Tissue required for diagnosis and clinical care was preserved, and a tumor sample and grossly normal tissue were flash frozen and stored at −80°C. Environmental controls from the operating room (open container of water left open during surgery, sterile gloved fingertip swirled in water, post-surgery gloved fingertip swirled in water) and pathology lab (container used to transport tissue from operating room to sterile hood, sterile dissecting tools rinsed in water) were collected and frozen as well.

### DNA extraction

Total DNA was extracted from the breast tissue, environmental controls, urine, and oral rinse pellets using PowerMag Microbiome RNA/DNA Isolation Kit according to the manufacturer’s protocol (MO BIO Laboratories Inc., Carlsbad, CA) with minor modifications as previously described [45]. Extraction and no-template controls consisting of reagents only were processed in parallel in an identical fashion.

### 16S rRNA gene sequencing

Bacterial 16S rRNA amplification and library construction was performed according to the 16S Metagenomic Sequencing Library Preparation guide from Illumina (Forest City, CA) with minor modifications. All beads, tubes, and nonenzymatic reagents were treated with UV light for at least 30 minutes prior to use [46]. In brief, 2 μl total DNA was amplified using primers F: (5′TCGTCGGCAGCGTCAGATGTGTATAAGAGACAGCCTACGGGNGGCWGCAG3′) and R: (5′GTCTCGTGGGCTCGGAGATGTGTATAAGAGACAGGACTACHVGGGTATCTAATCC3′), targeting the 16S V3 and V4 region (Illumina) [47] under the following conditions: 95°C for 3 minutes, followed by 25 cycles of 95°C for 30 seconds, 54°C for 30 seconds for breast samples or 55°C for 30 seconds for urine and oral rinse samples, 72°C for 30 seconds, and a final extension of 72°C for 5 minutes. For urine and oral rinse samples, the resulting 16S rDNA amplicons were cleaned with Ampure XP beads (Beckman Coulter, Inc., Brea, CA). For breast samples, the PCR product showed nonspecific bands on a 1% agarose gel. Thus, all breast sample 16S rDNA amplicons were run out on a 1% agarose gel, size selected at 450-500 bp, and gel purified using Zymoclean DNA Gel Recovery kit (Zymo, Orange, CA). Purified amplicons underwent a second round of PCR to add Nextera indices (Illumina) in half reaction volumes with 2.5 μl of sample. A second round of Ampure XP clean-up was performed and resulting libraries were quantified with Quantiflour dsDNA system (Promega, Madison, WI). After calculating molarity, 5 uL of each sample (diluted to 4 nM) were pooled into sequencing libraries. Libraries were validated on a Bioanalyzer DNA 1000 chip (Agilent, Santa Clara, CA) to verify size and sequenced on the Illumina Miseq with V3 reagent kit at the Case Western Reserve University Genomics Core.

### Bioinformatic analysis

Paired-end reads at 250 bp in length were merged using FLASH [48] with read length set to 250, fragment length set to 460, and standard deviation set to 46. Filtration of poor quality reads (Phred quality score < 20), subsampled open reference operational taxonomic unit (OTU) picking [49] against Greengenes (version 13.8) [50, 51] at 97% similarity threshold using UCLUST [52], alignment with PyNAST [53], phylogenetic tree construction using FastTree (version 2.1.3) [54], and subsequent computation of alpha (Shannon diversity index) [55] and beta diversity measures (unweighted Unifrac distances) [24] was performed using QIIME (version 1.9.1) [56]. Prior to PCoA analysis on weighted Unifrac distances of oral rinse and urine samples, OTU tables were normalized using the DESeq2 algorithm (implemented within the QIIME pipeline), which was originally developed for RNA-seq data, and models counts with a negative binomial mixture model [57, 58]. Breast tissue samples were rarefied to 60 reads instead of being normalized due to insufficient read counts for comparisons of both alpha and beta diversity. Sensitivity analyses with weighted UniFrac distances were also conducted, and were not reported unless discordant with findings based on unweighted UniFrac distances.

### Statistics

Two-sided Student’s T-test and Fisher’s exact test were used to compare continuous and categorical demographics/clinical factors, respectively, between cancer and non-cancer patient samples. Two-sided Student’s T-test was used to compare Shannon index averaged between 10 rarefactions per sample. Distance matrices were compared using PERMANOVA [59], a method which relies on F-tests based on sequential sums of squares derived from 1000 permutations on the weighted and unweighted UniFrac distance matrices, with the null hypothesis that there is no difference in community structure between groups. To compare relative abundances of taxa between different categorical variables, we used a nonparametric Wilcoxon rank-sum or Kruskal-Wallis test. To examine correlations between relative abundances of taxa and continuous variables, we used a bivariate analysis with log-transformed response abundances and a linear fit.

Relative abundances of each taxon for each sample are listed in Supplementary Table 3. In order to account for the confounding factor of menopausal status in the analysis of the urine microbiome, instead of using Wilcoxon rank-sum or Kruskal-Wallis, taxa summaries generated in QIIME [56] were reformatted for input into LEfSe [60] via the Huttenhower Lab Galaxy Server [61–63], where menopausal status was used as a subclass, and cancer vs. non-cancer as the overarching class. This ensured that any taxa flagged as significantly different between cancer and non-cancer urine samples by LEfSe’s algorithm were also different within the peri/post-menopausal and pre-menopausal subclasses. Taxa represented in fewer than 10% of total samples in the group were discarded. This algorithm performed nonparametric statistical testing of whether individual taxa differed between the class cancer vs. non-cancer, and the subclass peri/postmenopausal vs. premenopausal, and ranked differentially abundant taxa by their linear discriminant analysis (LDA) log-score [60]. Differentially abundant taxa that were statistically significant using an alpha of 0.05 and exceeded an LDA log-score of 2 were visually represented on cladograms and box plots, with *p*-values listed in Supplementary Table 4.

All statistical tests were two-sided, with *p*-value of less than 0.05 considered statistically significant. All analyses were conducted and graphs created in JMP Pro 12 (SAS Institute Inc., Cary, NC) or R version 3.2.2 (package *lattice*) [64]. The cladogram was created using GraPhlAn on Galaxy [61, 65].

## SUPPLEMENTARY MATERIALS FIGURES AND TABLES









## Abbreviations

ER: estrogen receptor
PCoA: principal coordinates analysis
OTU: operational taxonomic unit
BMI: body mass index
H: Shannon index
N: observed operational taxonomic unit count
