# Supplementary material for: Breast tissue, oral and urinary microbiomes in breast cancer
Source: Oncotarget. 2017 Aug 14;8(50):88122–38. doi: 10.18632/oncotarget.21490 (PMC5675698; doi:10.18632/oncotarget.21490)
Supplement: Supplementary file 1 [file oncotarget-08-88122-s001.pdf]

# Breast tissue, oral and urinary microbiomes in breast cancer

## SUPPLEMENTARY MATERIALS

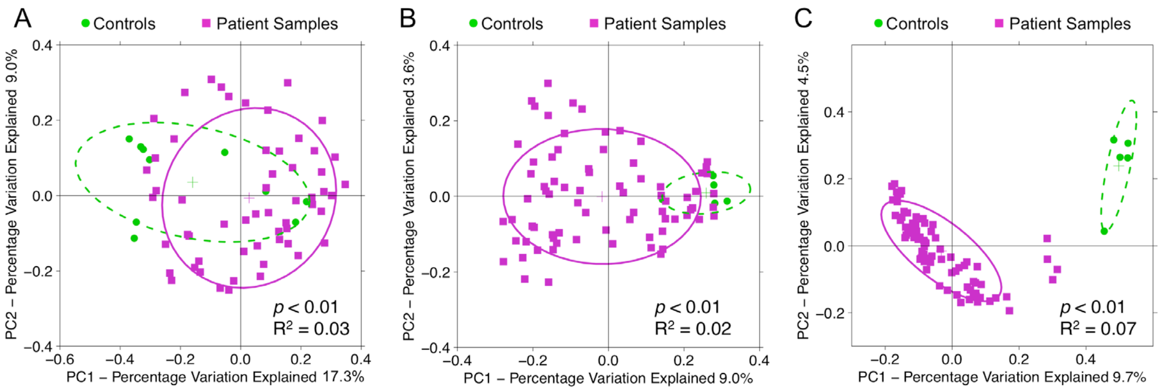


**Supplementary Figure 1: Principal coordinates analysis plots on unweighted UniFrac distances of patient and control samples.** Overall oral microbiomic diversity of patient and control (environmental, extraction, and no template) samples as represented by the first two principal coordinates on principal coordinates analysis of unweighted UniFrac distances. Each point represents a single sample, with plus sign and ellipses representing the fitted mean and 68% confidence interval of each group respectively. In **(A)** breast tissue, **(B)** urine, and **(C)** oral wash, control samples (green) separated significantly from patient samples (magenta). In breast tissue, although the difference between groups was statistically significant, there were several control samples that were similar to patient samples. For this reason, we only reported on taxa significantly increased in patient over control samples in the results.

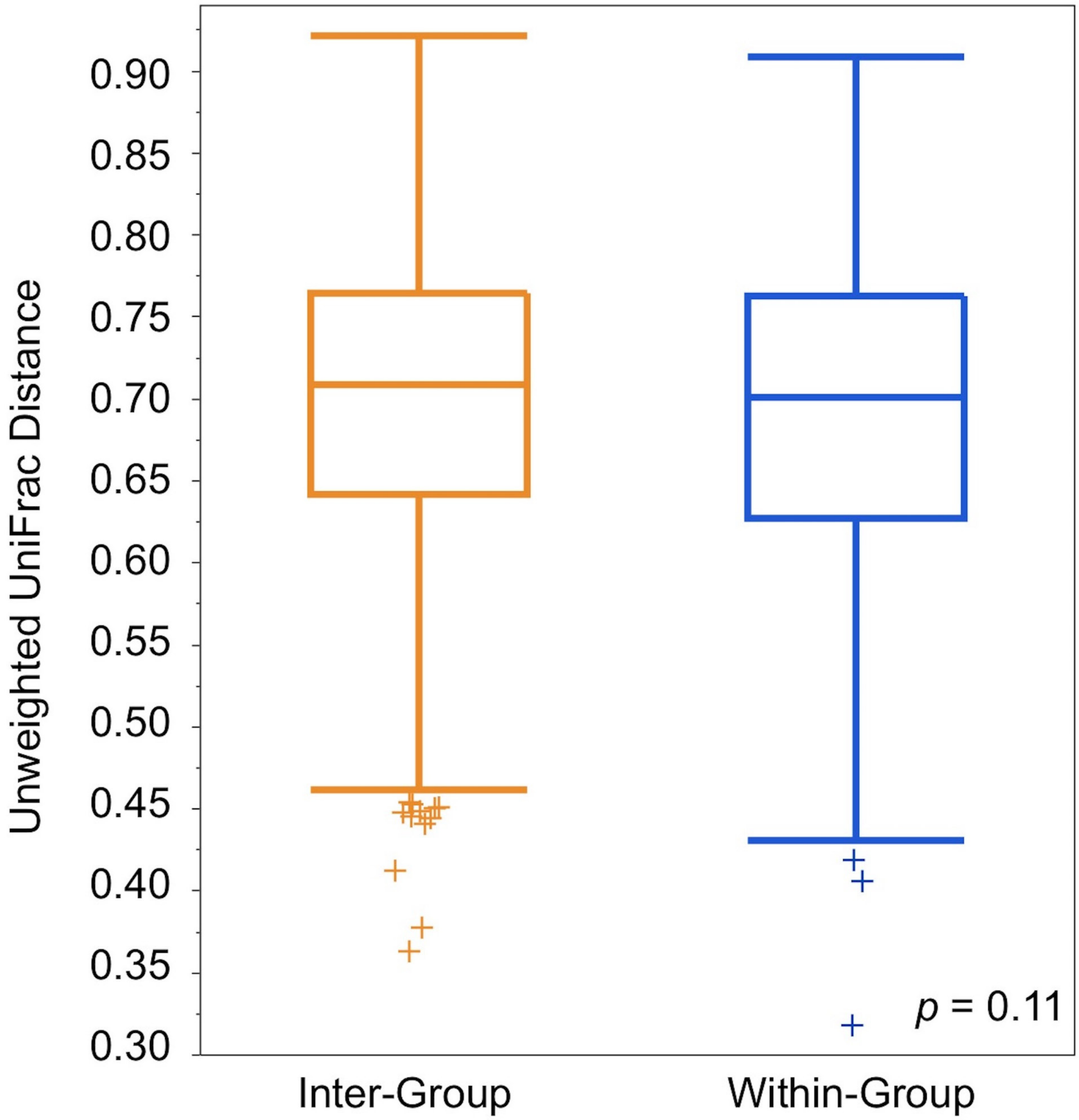


**Supplementary Figure 2: Distribution of inter-group and within-group unweighted UniFrac distances for cancer and non-cancer patient breast microbiome samples.** Boxplots representing distribution of unweighted UniFrac distances between cancer and non-cancer patient breast tissue samples (orange) as compared to among cancer or non-cancer patient samples (blue), indicating that samples within the same group (e.g. cancer or non-cancer) are not significantly more similar to each other than samples across or between groups (cancer vs. non-cancer).

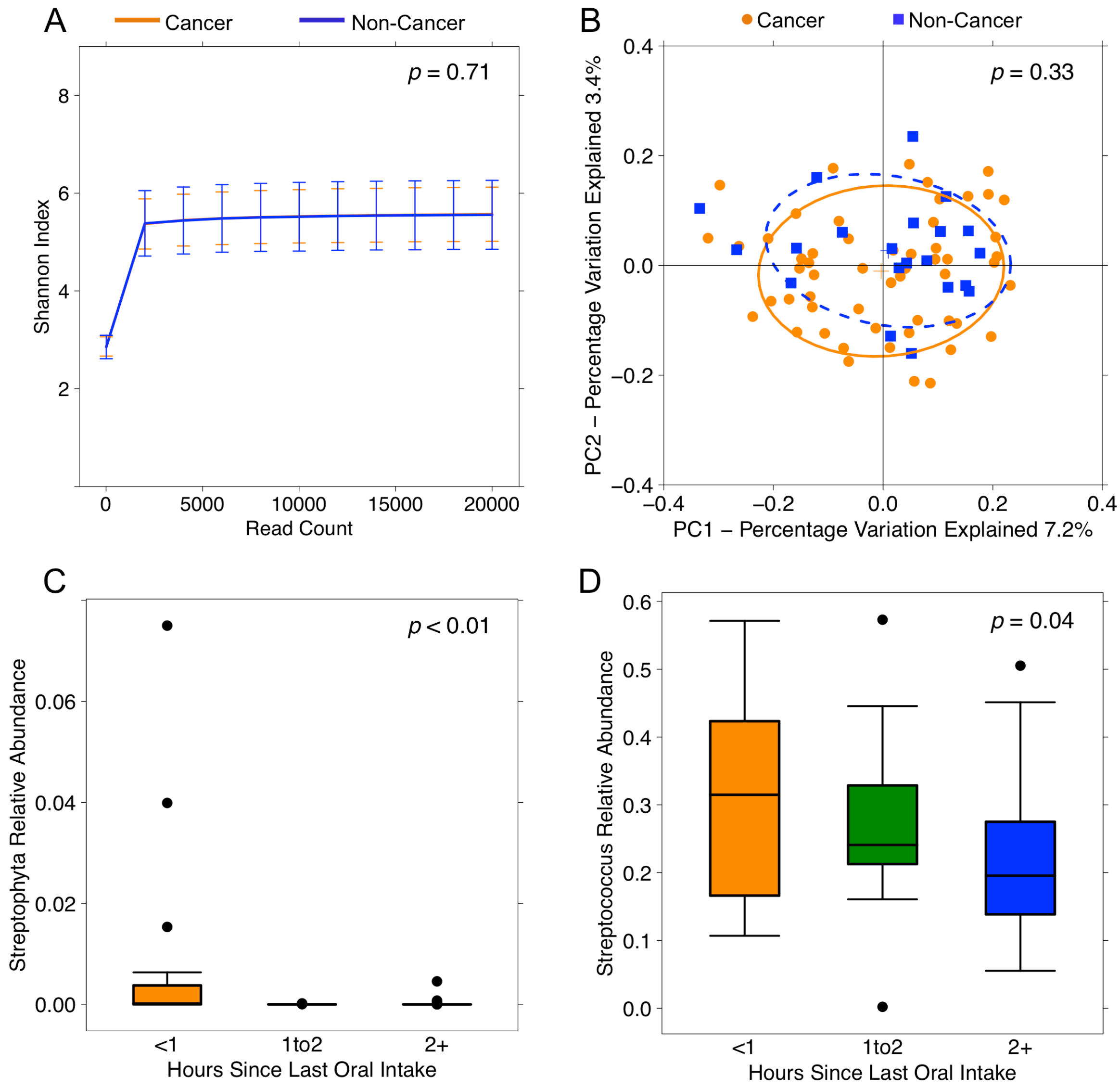


**Supplementary Figure 3: Oral rinse samples of cancer and non-cancer patients.** Oral rinse samples of cancer and non-cancer samples are largely similar on alpha and beta diversity metrics. Any differential taxon abundances can be explained by time since last oral intake. **(A)** Rarefaction curves of Shannon diversity index up to 20,000 reads were similar in cancer (orange) and non-cancer (blue) samples. **(B)** Principal coordinates analysis plot on unweighted UniFrac distances show no significant differences between cancer (orange) and non-cancer (blue) oral rinses. Time since last oral intake was significantly correlated with relative abundances of **(C)** Streptophyta (presumably chloroplasts from undigested plant material), and **(D)** Streptococcus as represented by box plots.

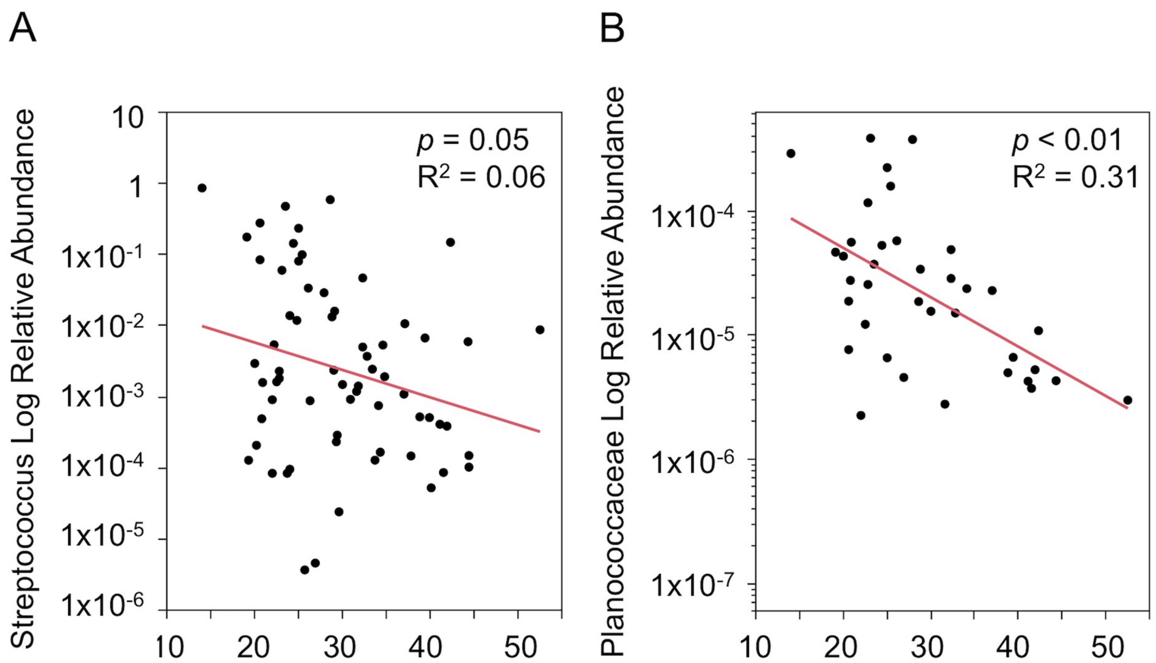


**Supplementary Figure 4: Relative abundances of Streptococcus and Planococcaceae by body mass index.** Dotplot and linear fit of log-transformed relative abundances of **(A)** *Streptococcus* and **(B)** Planococcaceae, demonstrating correlation with body mass index by bivariate analysis, which confounds interpretation of these taxa in cancer status.

**Supplementary Table 1: Clinical-pathologic characteristics of study patients with breast cancer**

| Variable | Cancer patients (N=57) |
|---|---|
| Presentation | |
| Symptomatic | 34 (60) |
| Mammogram Screening | 23 (40) |
| Tumor Size (cm) | 2.5 [1.9 – 3.7] |
| Pathologic T-stage | |
| 1 | 22 (39) |
| 2 | 30 (53) |
| 3 | 5 (9) |
| Multifocal | 15 (26) |
| Histologic Subtype | |
| Invasive Ductal Carcinoma | 41 (72) |
| Invasive Lobular Carcinoma | 9 (16) |
| Other | 7 (12) |
| Histologic Grade | |
| 1 | 17 (30) |
| 2 | 17 (90) |
| 3 | 23 (40) |
| Hormone Receptor-Positive | 50 (88) |
| *HER2* Amplified | 9 (16) |
| Lymphovascular Invasion* | 16 (30) |
| Node Positive | 23 (40) |

Values are presented as medians [Q1-Q3] or number (percent).
*Data is missing for lymphovascular invasion in 2 samples. In these cases, percentages are calculated from denominator of samples with known data.

**Supplementary Table 2: Demographics and clinical-pathologic characteristics by patient**

See Supplementary File 1

**Supplementary Table 3: Taxa tables for breast tissue, oral rinse, and urine specimens**

See Supplementary File 2

**Supplementary Table 4: Taxa identified as differentially abundant by Wilcoxon rank-sum testing**

See Supplementary File 3
